# Low Long Terminal Repeat (LTR)-Retrotransposon Expression in Leaves of the Marine Phanerogam *Posidonia Oceanica* L.

**DOI:** 10.3390/life10030030

**Published:** 2020-03-24

**Authors:** Alberto Vangelisti, Flavia Mascagni, Gabriele Usai, Lucia Natali, Tommaso Giordani, Andrea Cavallini

**Affiliations:** Department of Agriculture, Food and Environmental Sciences, University of Pisa, Via del Borghetto 80, I-56124 Pisa, Italy; albertovangelisti@libero.it (A.V.); flavia.mascagni@unipi.it (F.M.); gabri.usai@hotmail.it (G.U.); lucia.natali@unipi.it (L.N.)

**Keywords:** Illumina RNA-seq, LTR-retrotransposons, *Posidonia oceanica*, retrotransposon expression, seagrasses

## Abstract

Seagrasses as *Posidonia oceanica* reproduce mostly by vegetative propagation, which can reduce genetic variability within populations. Since, in clonally propagated species, insurgence of genetic variability can be determined by the activity of transposable elements, we have estimated the activity of such repeat elements by measuring their expression level in the leaves of plants from a Mediterranean site, for which Illumina complementary DNA (cDNA) sequence reads (produced from RNAs isolated by leaves of plants from deep and shallow meadows) were publicly available. Firstly, we produced a collection of retrotransposon-related sequences and then mapped Illumina cDNA reads onto these sequences. With this approach, it was evident that *Posidonia* retrotransposons are, in general, barely expressed; only nine elements resulted transcribed at levels comparable with those of reference genes encoding tubulins and actins. Differences in transcript abundance were observed according to the superfamily and the lineage to which the retrotransposons belonged. Only small differences were observed between retrotransposon expression levels in leaves of shallow and deep *Posidonia* meadow stands, whereas one *TAR/Tork* element resulted differentially expressed in deep plants exposed to heat. It can be concluded that, in *P. oceanica,* the contribution of retrotransposon activity to genetic variability is reduced, although the nine specific active elements could actually produce new structural variations.

## 1. Introduction

Seagrasses are marine phanerogams, i.e., monocotyledonous species belonging to Alismatales, which have colonized the marine environment in different regions. Seagrasses have important ecological functions in maintaining the marine environment, preventing shoreline erosion, providing marine water oxygenation, playing a nursery role for many species [1,2]. A global, progressive decline of seagrass meadows has been observed throughout the years in many areas of the world [3,4] because of human activities [5] and climate change [6], which favor the propagation of introduced chlorophytes, such as *Caulerpa taxifolia* in the Mediterranean Sea [7]. For these reasons, seagrass meadows are included among the most threatened ecosystems on earth [8,9].

Marine phanerogams reproduce both sexually and clonally [10]. Clonal propagation may offer ecological advantages in terms of resource/risk sharing among ramets within genotypes, especially for long-living species [11]. However, it is generally acknowledged that sexual reproduction is important to produce and maintain the genetic variability of a population. Within a species, populations with greater biodiversity generally exhibit greater productivity and recover better from disturbance than genetically uniform populations [12,13,14,15]. On the contrary, widespread vegetative propagation can determine a progressive reduction of genetic variability of the population and, consequently, of its potential to cope with environmental changes. Such potential would rely only on epigenetic mechanisms of gene regulation that facilitate and optimize phenotype variation [11]. The decline of seagrass meadows has been also related to the widespread vegetative propagation occurring in many regions of the world, such as the Mediterranean Sea [16]. For example, in many Mediterranean areas, *Posidonia oceanica*, a marine phanerogam endemic of this region, only seldom experiences flowering and sexual recruitment [17,18].

In clonally propagated species, insurgence of genetic and epigenetic variability can be determined by the activity of transposable elements. Transposable elements, or transposons, are mobile DNA sequences, abundant in every eukaryotic genome, which can change their chromosomal location through a mechanism, called transposition, operated by enzymes encoded by the transposon itself. Transposons can be classified as class I elements, also named retrotransposons (REs), or class II elements, the DNA transposons, depending on their transpositional mechanism [19]. In particular, class I elements transpose through a replicative mechanism consisting in the transcription of an RNA intermediate, followed by its reverse-transcription to cDNA, and by the insertion of the complementary DNA (cDNA) in another genomic locus [19]. Such a replicative mechanism, called retrotransposition, has allowed REs to colonize eukaryotic genomes, often accounting for hundreds of thousands of copies [20,21].

Based on the presence of long terminal repeats (LTRs) at their ends, retrotransposons are classified as LTR- and non-LTR-REs. In the LTR-REs, which are the most abundant in higher plants, LTRs contain promoter elements, polyadenylation signals, and enhancers, which regulate the expression of the RE [22]. The sequence between the two LTRs represents the coding portion of the element and includes the *Gag* and the *Pol* domains. *Gag* encodes virus-like particles, while *Pol* encodes the enzymes necessary for processing RE transcripts, as a reverse transcriptase, an integrase, a RNase H, and a protease [22]. Other structural features of LTR-REs are a primer binding site and a poly-purine tract, both involved in the RE replication [22].

LTR-REs can be classified into two major superfamilies, *Gypsy* and *Copia* [19], depending on the order of the enzyme-coding regions within the Pol domain. Superfamilies, on their turn, are subdivided into a number of lineages in relation to their structure and sequence [23,24,25,26,27,28,29].

Retrotransposition has determined (and is still determining) extensive variations in the genome structure of eukaryotes, even within one and the same species [30,31]. Among the consequences of retrotransposition, it has been observed that RE-related structural variations often produce changes in the expression regulation of genes adjacent to the newly inserted element, with consequent phenotypic changes that are subjected to selection and can contribute to the adaptation of a species to changes in the environment [32,33,34,35]. Retrotransposon-related changes in gene regulation can also rely on changes in the epigenetic settings of the genome and chromatin organization. Such epigenetic changes allow organisms to fine-tuning phenotypes on shorter time scales than common genetic variations [36].

The aim of this work was evaluating the potential activation of retrotransposition events in *P. oceanica* by measuring the transcription of LTR-RE sequences, which represents the first step of the retrotransposition process. Unfortunately, because of the large size of *P. oceanica* genome (more than 5 Gbp [37]), which would require very high sequencing coverage to obtain large sequenced scaffolds, no full-length LTR-REs are available of this species. For this reason, we conducted a meta-analysis using a library of LTR-RE fragments previously identified in *P. oceanica* [38]. The transcription of these LTR-RE sequences was estimated by mapping the Illumina cDNA read libraries, available in a public repository, produced by Marin-Guirao et al. [39] onto a reference transcriptome from plants of deep and shallow *Posidonia* meadow stands.

## 2. Materials and Methods

### 2.1. P. oceanica DNA and cDNA Illumina Reads Collection

Illumina reads of *P. oceanica* were downloaded from Sequence Read Archive (SRA; https://www.ncbi.nlm.nih.gov/sra, see the Appendix A). The accession numbers of the sequences used in these analyses were PRJNA295148 for DNA sequences [38] and PRJNA353749 for cDNA sequences [39]. cDNA sequences were isolated from plants of a meadow off Isla Grosa (Spain), transplanted in individual tanks, and subjected to different treatments [39]. Only cDNA sequences isolated from control specimens were used.

DNA paired-end reads of 100 bp length were collected, overall quality was checked by FastQC (v. 0.11.5 [40]) and improved using Trimmomatic (v. 0.38 [41]) by removing adapter content and low quality reads with the following parameters: ILLUMINACLIP, SLIDINGWINDOW:4:20, CROP:93. Organellar reads were discarded mapping onto chloroplast and mitochondrion sequences of a closely related species, *Zostera marina*, available on National Center for Biotechnology Information (NCBI) online repository (https://www.ncbi.nlm.nih.gov/). Mapping was run on CLC Genomics Workbench (v. 9.5.3) with length fraction = 0.5 and similarity fraction = 0.8. Only the unmapped reads were retained.

Concerning cDNA reads, twenty-four paired-end libraries from leaves of plants from deep and shallow *P. oceanica* meadows, before and after 5 days of heat treatment (32 °C) treatment (six replicate each) were used [39]. Reads were improved in quality using Trimmomatic; due to quality of the reads, parameters for trimming were changed compared to DNA libraries previously described. Parameters were set as following: ILLUMINACLIP, HEADCROP:12, SLIDINGWINDOWS:4:20, MINLEN:88. Possible ribosomal RNA traces were removed mapping the reads on rDNA of *P. oceanica*, available on SILVA repository [42] using CLC Genomics Workbench, as described for organellar filtering.

### 2.2. Retrotransposons Sequence Set of P. oceanica

Retrotransposon sequences of *P. oceanica* (from a meadow off Antignano, Livorno, Italy) were collected from the set of repeated sequences assembled [38], retaining only repeats annotated as LTR-REs (see the Appendix A). These sequences were further annotated as belonging to retrotransposable elements depending on their alignment (by BLASTn, with default parameters) to a custom collection of full-length LTR-REs of a closely related seagrass, *Zostera marina*. For this species, the genome sequence is now available [43]. We identified full-length LTR-REs of *Z. marina* using the tool LTRharvest v. 1.5.10 [44] with stringent parameters (minlenltr = 100; maxlenltr = 6000; mindistltr = 1550; maxdistltr = 25,000; mintsd = 5; maxtsd = 5; similar = 85; vic = 10) and used them to verify the similarity of *P. oceanica* sequences to LTR-REs. In addition, we also annotated *P. oceanica* sequences by BLASTn analysis against the RiTE database of rice repeated elements [45].

Annotation of *Posidonia* sequences was also performed submitting sequences to InterProScan (v. 5.33-71.0 [46]) against the PFAM database [47] to ascertain the occurrence of LTR-RE protein domains.

For each LTR-RE sequence, the genomic abundance was assessed by aligning DNA reads of *P. oceanica* (see above) and counting the number of matching reads, using CLC Genomics Workbench (v. 9.5.3.) with the following parameters: length fraction = 0.9, similarity fraction = 0.9, mismatch penalty = 1, gap penalty = 1.

### 2.3. Retrotransposons Expression Analysis

Libraries of cDNA from control and heat-treated deep and shallow *P. oceanica* meadow stands (six replicates each) were used to analyze retrotransposon expression. As reference, we used an available de novo transcriptome of *P. oceanica* (obtained using leaves of plants from a meadow located in Stareso, Corse, France [48] to which were added the retrotransposon sequences [38].

Since de novo transcriptomes generally include LTR-RE sequences, it was necessary to exclude these sequences from the de novo transcriptome of *P. oceanica*. First, the LTR-RE sequence set was aligned to the de novo transcriptome by using CLC Genomics Workbench (similarity fraction = 0.9, length fraction = 0.9, mismatch penalty = 1, gap open penalty = 1) in order to select transcripts showing similarity to LTR-REs. Then, such transcripts were annotated using the NCBI non-redundant (nr) database (http://blast.ncbi.nlm.nih.gov/Blast.cgi) and sequences identified as retrotransposons were excluded, while the other sequences were maintained in the transcriptome. The transcriptome, deprived of all sequences putatively encoding LTR-RE-related proteins, was added to the retrotransposon set of sequences [38] and used as reference transcripts library.

High quality paired-end reads obtained from cDNA libraries, 88 nucleotide in length each, were aligned onto the reference transcripts library using CLC Genomics Workbench (similarity fraction = 0.9, length fraction = 0.9, mismatch penalty = 1, gap open penalty = 1). Sequences encoding actins and tubulins were used as reference genes.

The number of mapped reads of each LTR-RE sequence were normalized calculating the number of mapped reads per million of reads used for mapping (MRxM).

Pairwise comparisons between deep and shallow meadows, and between heat-treated and control plants, were performed by Baggerley’s statistical test, exploiting proportion comparison on t-test weighted by beta-distribution [49]. A sequence was considered differentially expressed when *p*-value < 0.05.

## 3. Results

### 3.1. Preparation of a Set of LTR-Retrotransposon Sequences of P. oceanica

Barghini et al. [38] assembled Illumina reads of *P. oceanica* and produced a whole-genome set of assembled sequences (*Po*WGSAS), made of 19,760 contigs, of which 4426 were annotated as related to LTR-retrotransposons. In the present experiments, we annotated *Po*WGSAS retrotransposon sequences by BLAST analysis against a custom database of LTR-retrotransposons of a seagrass related to *P. oceanica*, *Zostera marina* (produced using LTRharvest at high stringency on the *Zostera* genome sequence) and against a public database of rice repeated elements (RiTE [45]). The annotation of *Po*WGSAS retrotransposon sequences was further assessed by verifying the occurrence of LTR-retrotransposon domains.

At the end of these analyses, the set of *P. oceanica* LTR-retrotransposon fragments included 180 sequences. No full-length elements were retrieved, as expected because of the low coverage (0.28) of the DNA-seq used for assembling the *Po*WGSAS [38].

**Figure 1:**
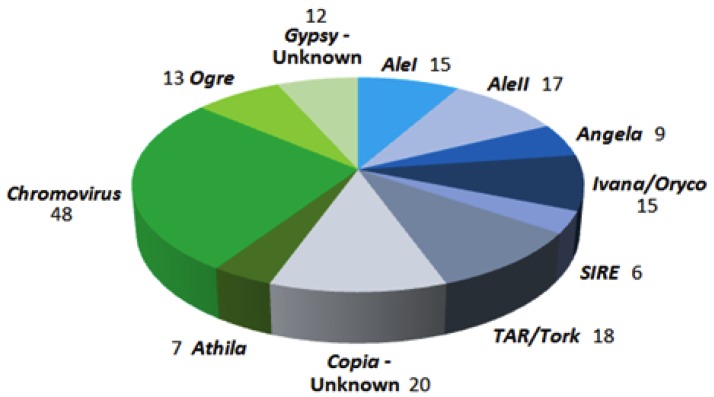
Number of sequences corresponding to different lineages of long terminal repeats (LTR)-retrotransposons (REs) in the set of *P. oceanica* collected sequences.

The composition of the collection of LTR-retrotransposon sequences of *P. oceanica* is reported in Figure 1. All main lineages of *Gypsy* and *Copia* superfamilies were represented. For 32 sequences, the superfamily only could be identified, 20 of the *Copia* and 12 of the *Gypsy* superfamily, respectively. As expected [38], the most represented lineage was *Gypsy-Chromovirus*, with 48 sequences.

### 3.2. Analyses of LTR-RE Expression in Deep and Shallow Plants In Vivo

The expression of the 180 LTR-retrotransposon fragments was analyzed using Illumina cDNA libraries of *P. oceanica*, obtained from leaves of plants taken from shallow or deep meadow stands in south-eastern coast of Spain [39], publicly available in the SRA database (see Materials and Methods). Mapping was performed on a sequence set composed of gene sequences of *P. oceanica* [48] (see Materials and Methods) and of the 180 retrotransposon fragments. Expression data are reported in Supplementary file #1.

**Figure 2:**
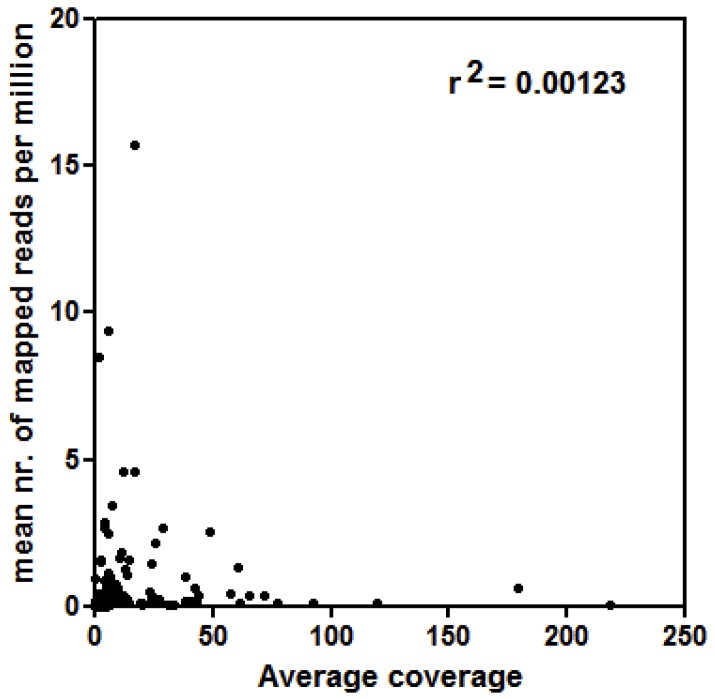
Relationship between expression values (mean number of mapped reads per million reads used for mapping) of each of the 180 LTR-RE sequences of *P. oceanica* and the respective abundance in the genome (indicated by the average coverage). The correlation coefficient (r^2^) is reported.

The in vivo expression of LTR-retrotransposons of *P. oceanica* was generally low: only a mean of 0.01% of reads of both shallow and deep plants, respectively, mapped onto the set of LTR-retrotransposon sequences. In order to exclude that the occurrence of LTR-RE sequences in cDNA libraries could be related to genomic DNA contamination, we compared the mean number of mapped reads per million of a LTR-RE sequence to its genomic abundance (Figure 2). The most abundant LTR-REs were barely expressed and, correspondingly, the most expressed elements were lowly represented in the *P. oceanica* genome.

**Figure 3:**
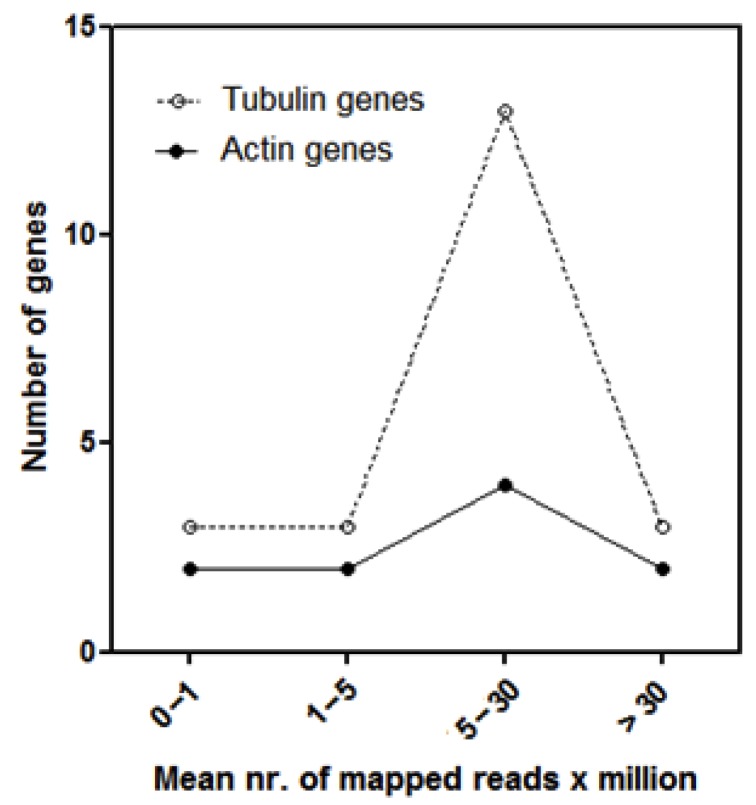
Number of genes encoding tubulin or actin, subdivided into four classes according to the respective expression level (mean number (nr.) of mapped reads per million reads used for mapping).

In order to establish the extent of the expression level of retrotransposon-related sequences, the transcription of different copies of actin- and tubulin-encoding genes, identified in the transcriptome of *P. oceanica* [48], was concurrently measured. The transcript abundance of 10 actin- and 22 tubulin-encoding genes in leaves of plants of shallow *Posidonia* meadow stands is reported in Figure 3. Although two actin- and two tubulin-encoding transcripts were highly expressed, as expected, the majority of both tubulin and actin genes were expressed at medium level (between 5 and 30 mapped reads x million, Figure 3).

**Figure 4:**
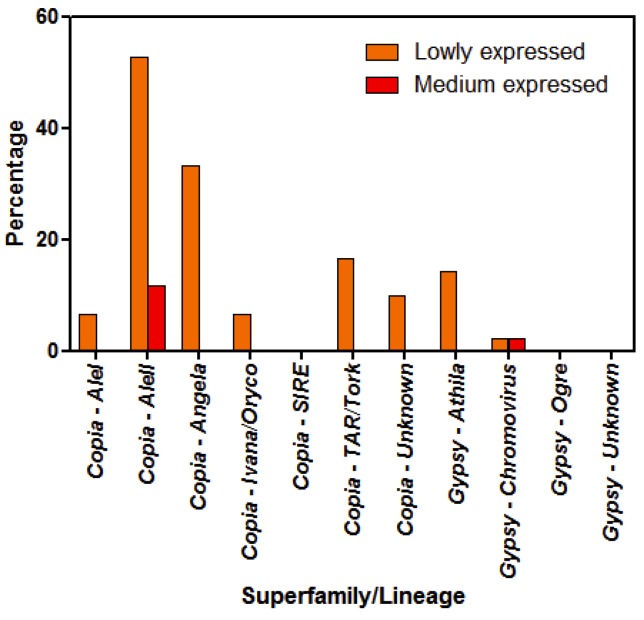
Percentage of lowly (one to five mapped reads per million) and medium expressed (>5 mapped reads per million) LTR-RE sequences in leaves of shallow and deep *P. oceanica* plants, calculated for each LTR-RE lineage.

Since one mapped read per million is often the threshold at which a sequence is considered as expressed [50,51], we established that LTR-RE showing a mean of one to five mapped reads per million were lowly expressed. Overall, only 24 over 180 sequences were considered as lowly expressed. Using a threshold of five mapped reads x million, only three sequences resulted medium transcribed. The percentages of sequences lowly and medium expressed for each LTR-RE lineage is reported in Figure 4.

*Copia* elements were apparently more transcribed than *Gypsy* ones (in total 21 vs. 3). Furthermore, it is worth noting that *AleII* lineage is by far the most transcriptionally active, being 52.9% of *AleII* sequences lowly expressed, and 11.8% medium expressed. On the contrary, *SIRE* sequences, *Ogre* sequences, and sequences of the *Gypsy* superfamily of which the lineage was not identified were never transcribed.

### 3.3. Differential LTR-Retrotransposon Expression Analyses

RNA sequencing (RNA-seq) analysis of differential expression between deep and shallow prairies and between heat-treated and control plants is reported in Supplementary material #1. Because of the generally low transcript abundance of *P. oceanica* retrotransposons, we report the analyses of the 24 elements showing low and medium transcription level (Table 1).

Table 1 shows that no elements resulted significantly over- or under-expressed between plants taken from shallow and deep meadow stands. Although for all elements differences were not significant, it is noteworthy that all elements but two (one *TAR/Tork* and one *Chromovirus* sequences) were more expressed in shallow than in deep meadow stands. Considering the reference genes (22 tubulin- and 10 actin-encoding), only one over 32 showed a significant difference in expression between shallow and deep meadows.

Comparing the expression of LTR-RE between heat-treated and control plants showed no differentially expressed element in heat-treated shallow plants and only one differentially expressed LTR-RE in deep plants. This element (Poc_contig_3029) belongs to the *TAR/Tork* lineage.

## 4. Discussion

In this work, we analyzed the expression of a number of LTR-RE related sequences of *P. oceanica*. Full-length elements would have been more suitable than LTR-RE sequence fragments for studying RE expression. As a matter of fact, in this species, full-length elements are not available. In this case, a useful strategy for analyzing the expression of REs can be to prepare a comprehensive library of repeated elements and then analyze the expression of these elements by mapping with Illumina cDNA reads, as it is usually done with genes in RNA-seq experiments [52].

We decided to use LTR-RE sequences assembled in a previous work [38]; however, in this work we further annotated LTR-RE sequences comparing them to RE collections of *Z. marina* and of rice, which were not available in 2015, and retained only sequences showing similarity to LTR-REs of these species. Therefore, it cannot be excluded that other LTR-REs, highly specific to *P. oceanica*, exist and are active.

The analyzed REs resulted generally lowly transcribed. The low transcript abundance of LTR-REs in plants has often been reported [51,53,54,55,56]. In certain species, increased expression in plants exposed to biotic or abiotic stresses was reported; however, the global level of expression remained low even during stressful treatments [57,58,59,60,61,62]. Our data indicated that 21/180 sequences were barely expressed and only three sequences were transcribed at levels comparable to those of the majority of actin- and tubulin-encoding reference genes. Low transcript abundance of repeated DNA sequences as LTR-REs might be related to genomic DNA contamination in the RNA-seq libraries, rather than to actual transcription of the related elements. However, our data showed that most expressed elements were the least abundant in the genome; hence, genomic DNA contamination can be ruled out.

Between LTR-RE superfamilies, *Copia* elements were slightly more expressed than *Gypsy* ones, especially considering the 24 lowly/medium transcribed REs. That *Copia* LTR-REs are more transcribed than *Gypsy* was somewhat expected, because this superfamily resulted less abundant than *Gypsy* in the genome of *P. oceanica* [38] and it is known that lowly redundant elements are generally less subjected to transcription inhibition by defense mechanisms of the cell [35]. On the other hand, many of the LTR-REs expressed in other species belong to the *Copia* superfamily [63].

Among lineages, *AleII* elements were more transcribed than the other LTR-RE lineages since 11 over 24 lowly/medium transcribed elements belonged to this lineage. LTR-RE lineage-depending expression was already reported in cotton [64]. In tobacco, both *Tnt*1 and *Tto*1 (induced by tissue culture) belong to the *TAR/Tork* lineage [29]. In sunflower, the most expressed elements belong to the *AleII* lineage [65], as in *P. oceanica*.

Interestingly, Barghini et al. [38] showed that *AleII* elements of *P. oceanica* are among the most uniform in sequence compared to other lineages, suggesting that *AleII* LTR-REs have been active in retrotransposition in recent evolutionary times. The present data suggest that *AleII* elements are still active. Similar data were reported in *Arabidopsis halleri gemmifera* [66].

We also compared the expression of medium expressed elements between deep and shallow *P. oceanica* meadow stands. In general, LTR-RE expression levels were slightly higher in leaves of shallow than of deep meadow stands; however, no sequences resulted significantly more transcribed in shallow than in deep plants, indicating that the depth of the meadow was only barely relevant to the LTR-RE expression.

Our data show that LTR-REs are barely expressed; hence, these elements should not significantly contribute to genetic variability in this species. On the other hand, it is not possible to know if, during *Posidonia* evolution, LTR-REs have actually produced genetic variability: the complete genome sequence of different *Posidonia* accessions would be necessary to estimate the contribution of such elements to variation in genome sequences. It is possible that LTR-RE insertions have occurred in some plants, in these cases the effect of the natural selection on the contribution of LTR-RE insertions to genome sequence variability should be taken in account [67].

Obviously, the expression of a LTR-RE is only a pre-requisite for its transposition. As a matter of fact, to complete the retrotransposition, the element, after having been transcribed, should be reverse-transcribed and inserted in a new site of the genome. Such a complete retrotranspositional process has been described only in a few studies: the expression of *Tnt*1 and *Tto*1, and of *Tos*17 in tissue cultures of *Nicotiana* and of rice, respectively, were followed by their subsequent insertion in the genome [68]. Complete retrotransposition of a *Copia* element was also reported in normally cultivated plants of sunflower [69]. These steps in retrotransposition are often counteracted by defense mechanisms of the cell, as for example RNA silencing [35,70,71]. Since the generally low expression of LTR-REs, it may be hypothesized that such defense mechanisms operate efficiently in *P. oceanica*. In this sense, it is possible that retrotransposon activity slightly contributes in producing new genetic variability in this seagrass, although even a single transcript could have an effect on the genome.

Although only one (of the *TAR/Tork* lineage) over 180 LTR-REs resulted significantly over-expressed in heat-treated than in control plants, it cannot be excluded that retrotransposon expression in meadows subjected to environmental constraints can increase, since environmental stresses often induce transposable element transcription, as observed in another seagrass, *Cymodocea nodosa*, under experimental acidification [72]. Such an activity might produce genetic and epigenetic variations, which could be selected in those environments. Further studies are necessary and are in progress to test this hypothesis.

## Figures and Tables

**Table 1:** ID code, superfamily, lineage, length, and mean transcript abundance of six biological replicates per treatment (mean number of mapped reads per million reads used for mapping) of 21 low- and 3 medium-expressed LTR-RE sequences in leaves of shallow and deep *P. oceanica* plants (*: *p* < 0.05).

ID	Superfamily	Lineage	Length (nt)	Mean Number of Mapped Reads Per Million	
				Deep	Shallow
Poc_contig_4694	*Copia*	*AleI*	237	1.68	2.04
Poc_contig_11919	*Copia*	*AleII*	303	1.04	1.16
Poc_contig_13155	*Copia*	*AleII*	350	0.96	1.31
Poc_contig_16252	*Copia*	*AleII*	292	1.49	1.50
Poc_contig_2925	*Copia*	*AleII*	265	1.47	1.74
Poc_contig_11953	*Copia*	*AleII*	264	2.39	2.53
Poc_contig_11947	*Copia*	*AleII*	233	2.29	2.73
Poc_contig_7641	*Copia*	*AleII*	258	2.46	2.91
Poc_contig_5573	*Copia*	*AleII*	235	2.70	3.06
Poc_contig_10207	*Copia*	*AleII*	364	3.25	3.56
Poc_contig_14966	*Copia*	*AleII*	330	8.64	10.07
Poc_contig_10239	*Copia*	*AleII*	434	14.39	17.02
Poc_contig_13500	*Copia*	*Angela*	326	0.90	1.24
Poc_contig_3616	*Copia*	*Angela*	223	1.57	1.62
Poc_contig_6783	*Copia*	*Angela*	279	4.39	4.74
Poc_contig_2856	*Copia*	*Ivana/Oryco*	429	1.77	2.58
Poc_contig_13693	*Copia*	*TAR/Tork*	152	1.29	1.20
Poc_contig_16069	*Copia*	*TAR/Tork*	312	1.47	1.75
Poc_contig_3029	*Copia*	*TAR/Tork*	395	3.77	5.34
Poc_contig_12140	*Copia*	*Unknown*	207	1.02	1.04
Poc_contig_14451	*Copia*	*Unknown*	176	1.40	1.47
Poc_contig_3356	*Gypsy*	*Athila*	828	2.28	3.11
Poc_contig_6648	*Gypsy*	*Chromovirus*	301	1.19	1.41
Poc_contig_16201	*Gypsy*	*Chromovirus*	260	9.11	7.91

## Supplementary Material

The following are available online at https://www.mdpi.com/2075-1729/10/3/30/s1, Supplementary file 1, RNA-seq analysis (xlsx file).

## Appendix A

Raw reads of Illumina sequencing are accessible at NCBI SRA under the accession numbers PRJNA295148 (genomic DNA) and PRJNA353749 (cDNA). The collection of LTR-RE-related sequences and their annotations are available at the repository sequence page of the Department of Agriculture, Food, and Environment of the University of Pisa (http://pgagl.agr.unipi.it/sequence-repository/).
